# Deactivation of the antiviral state by rabies virus through targeting and accumulation of persistently phosphorylated STAT1

**DOI:** 10.1371/journal.ppat.1010533

**Published:** 2022-05-16

**Authors:** Gayathri Manokaran, Michelle D. Audsley, Haruka Funakoda, Cassandra T. David, Katherine A. Garnham, Stephen M. Rawlinson, Celine Deffrasnes, Naoto Ito, Gregory W. Moseley

**Affiliations:** 1 Department of Microbiology, Biomedicine Discovery Institute, Monash University, Melbourne, Australia; 2 Laboratory of Zoonotic Diseases, Joint Department of Veterinary Medicine, Faculty of Applied Biological Sciences, Gifu University, Gifu, Japan; Thomas Jefferson University - Center City Campus: Thomas Jefferson University, UNITED STATES

## Abstract

Antagonism of the interferon (IFN)-mediated antiviral state is critical to infection by rabies virus (RABV) and other viruses, and involves interference in the IFN induction and signaling pathways in infected cells, as well as deactivation of the antiviral state in cells previously activated by IFN. The latter is required for viral spread in the host, but the precise mechanisms involved and roles in RABV pathogenesis are poorly defined. Here, we examined the capacity of attenuated and pathogenic strains of RABV that differ only in the IFN-antagonist P protein to overcome an established antiviral state. Importantly, P protein selectively targets IFN-activated phosphorylated STAT1 (pY-STAT1), providing a molecular tool to elucidate specific roles of pY-STAT1. We find that the extended antiviral state is dependent on a low level of pY-STAT1 that appears to persist at a steady state through ongoing phosphorylation/dephosphorylation cycles, following an initial IFN-induced peak. P protein of pathogenic RABV binds and progressively accumulates pY-STAT1 in inactive cytoplasmic complexes, enabling recovery of efficient viral replication over time. Thus, P protein-pY-STAT1 interaction contributes to ‘disarming’ of the antiviral state. P protein of the attenuated RABV is defective in this respect, such that replication remains suppressed over extended periods in cells pre-activated by IFN. These data provide new insights into the nature of the antiviral state, indicating key roles for residual pY-STAT1 signaling. They also elucidate mechanisms of viral deactivation of antiviral responses, including specialized functions of P protein in selective targeting and accumulation of pY-STAT1.

## Author summary

Following viral infection, the host activates multiple antiviral defenses. The ability of viruses to overcome these defenses is critical to disease. The earliest antiviral response involves the production of interferon messenger molecules. Interferons act on infected cells to inhibit viral proliferation, as well as on non-infected cells to establish an antiviral state before infection and so limit viral spread through the host organism. Many strategies used by viruses to overcome the former are well understood, but mechanisms important to the latter, and their importance to disease, are less well defined. In this study, we investigated how rabies virus overcomes a pre-established antiviral state in target cells. We found that the capacity to disable the antiviral state correlates with the ability to cause disease, and involves binding of a viral protein to cellular signaling proteins, which our data indicate are responsible for the maintenance of a prolonged antiviral state. This advances our understanding of antiviral responses, and identifies a key step in lethal infection by rabies virus that causes approximately 60,000 human deaths per year. The findings may contribute to new approaches for the development of vaccines or antivirals.

## Introduction

The principal response of cells to viral infection is activation of the type-I interferon (IFN-I) system. Following detection of virus, IFN-I is induced and released from infected cells and signals in autocrine (feedback to the infected cell) or paracrine (signaling to neighboring cells) fashion, by binding to the type-I IFN receptor (IFNAR) [1,2]. This results in phosphorylation of signal transducers and activators of transcription (STAT)1 and STAT2 [1,2] at conserved tyrosines (Y701 in STAT1), generating pY-STATs. In a complex with IRF9, pY-STAT1/2 accumulate in the nucleus and bind to IFN-stimulated response elements (ISREs), activating hundreds of IFN-regulated genes (IRGs) including antiviral genes; this establishes an intrinsic cellular antiviral state, and facilitates the development of an adaptive immune response [1,2].

Following IFN activation of cells, STAT1 phosphorylation occurs rapidly, reaching a peak of pY-STAT1 within c. 30–60 min, during which pY-STAT1 accumulates in the nucleus to activate IRGs. pY-STAT1-DNA binding and transcriptional activation is followed by release of STATs from the DNA, dephosphorylation by nuclear phosphatases, and return to the cytoplasm [3–14]. Further phosphorylation/dephosphorylation cycles can support IRG regulation until IFN signaling is turned off. Thus, STAT1 appears to dynamically monitor IFNAR activity [15]. Peak pY-STAT1 levels and nuclear accumulation are typically lost within several hours, which is associated with depleted IRG expression [3,5–10,12,13,16–19]. Regulatory proteins including phosphatases and SOCS proteins turn off the IFN response, with several of these being IRGs that form negative feedback pathways [3,5].

These mechanisms cooperate to produce an appropriate response and avoid damage through excessive IFN signaling/IRG activity [3,10]. Nevertheless, following initial stimulation, the antiviral state can persist for extended periods, presumably to support viral clearance [10,20]. pY-STAT1 is required to establish an antiviral state [21] but is rapidly depleted by dephosphorylation, so the extended antiviral state is thought to involve unphosphorylated (U-) STAT1. As STAT1 is an IRG [9,10], U-STAT1 levels are elevated following IFN stimulation, and appear to induce prolonged expression of specific IRG subsets. This is proposed to maintain IRGs important to virus clearance, while depleting ‘early’ pY-STAT1-dependent IRGs that are potentially damaging if expressed for extended periods [9,10].

Viral subversion of the IFN response is central to infection and pathogenesis [1,2,22] and is mediated by viral IFN-antagonist proteins, which are often multifunctional and target several stages of the response (IFN induction, IFN signaling and IRG effector functions) *via* numerous mechanisms [22–25]. The ability of many viruses to block IFN induction [22] raises the question of why IFN signaling and effector stages are also inhibited. This likely relates to ‘leakiness’ in antagonism, so that some IFN is still released from infected cells, necessitating inhibition of pY-STAT activated by autocrine signaling. Importantly, paracrine signaling also activates non-infected cells to establish a protective antiviral state [1,2]. Furthermore, immune cells such as plasmacytoid dendritic cells, which are often not productively infected, produce IFNs during infections, resulting in paracrine signaling [26–29]. Thus, progeny virus must overcome an established antiviral state in target cells to spread within the host. However, analyses of viral IFN antagonism often use systems in which infection or viral protein expression is established prior to IFN treatment (e.g. [8,16,17,30–34]); while this provides important information on antagonistic mechanisms, it essentially mimics an autocrine response. As a result, for many viruses, knowledge on the mechanisms used to overcome a preexisting antiviral state, and their significance to pathogenesis, remains limited.

The main IFN antagonist of RABV is P protein [24], which can suppress IFN induction by blocking IRF3/IRF7 phosphorylation [35,36], and can antagonize signaling by STATs *via* physical interaction [8,16,17,25,30–32,37,38]. P protein also interacts with the IRG PML (promyelocytic leukemia) protein [39], and so may affect IRG functions. The P protein C-terminal domain binds directly to STAT1 but, in contrast to many other antagonists [22], does not affect STAT1 expression or tyrosine phosphorylation. Rather, P protein effects nuclear exclusion of STAT1 *via* a strong nuclear export sequence (N-NES) in the N-terminus of full-length P protein, and inhibits DNA interaction [8,16,17,25,30,40–43]. Intriguingly, efficient P-protein-STAT1 interaction is dependent on IFN-treatment, such that P protein appears to antagonize pY-STAT1 selectively [16,30,32]. P protein-pY-STAT1 binding also inhibits dephosphorylation so that in cells pre-infected by virus or expressing P protein, IFN treatment causes a rapid cytoplasmic accumulation of pY-STAT1 that persists at high levels for extended periods [8,16,17]. This is suggested to result from impaired nuclear accumulation and interaction with nuclear phosphatases [8,16,17]. Thus, pY-STAT1 recycling is impaired as part of P protein’s inhibitory mechanism [16].

**Fig 1:**
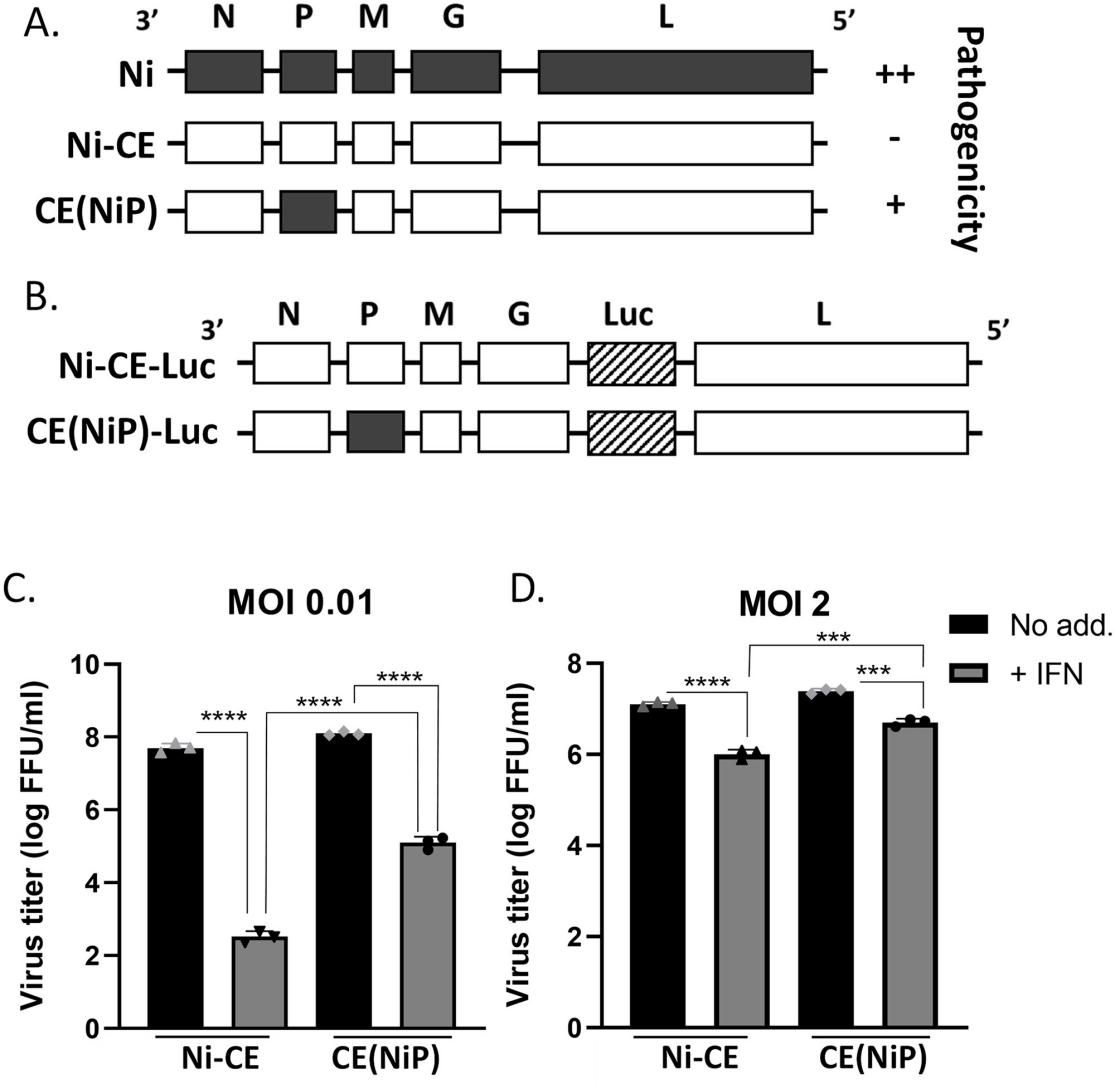
IFN sensitivity of Ni-CE and CE(NiP) viruses is dependent on MOI. (A,B) Schematic representation of genomes of Ni, Ni-CE and CE(NiP) viruses with relative pathogenicity (A), and of viruses containing the luciferase (Luc) gene (B). (C) SK-N-SH cells infected with the indicated virus at MOI of 0.01 or MOI of 2 were incubated in media containing IFN (+ IFN) or without IFN (No add.) at 500 U/ml for 3 or 2 days, respectively, before collection of supernatant and titration (mean with SD, n = 3). ***, p ≤ 0.001; ****, p ≤ 0.0001.

The importance of P protein-mediated STAT1 antagonism in the pathogenesis of RABV was revealed using the pathogenic *Nishigahara* (Ni) RABV strain that causes lethal infection in mice [25,44,45], and an attenuated Ni-derived strain (Ni-CE), which is defective in antagonism of STAT1 signaling, and non-pathogenic in mice. Substitution of the Ni P gene (which encodes P protein) into Ni-CE generated the CE(NiP) strain, in which a pathogenic phenotype was restored (Fig 1A), correlating with potent IFN/STAT-antagonistic function of Ni P protein compared with Ni-CE P protein, in which antagonistic function is strongly impaired [24, 45]. Ni-CE P protein can bind to STAT1, but is defective for nuclear export due to substitutions in the N-NES, and so cannot efficiently inhibit pY-STAT1 nuclear localization [24,25]. Thus, P protein is a pathogenesis factor, dependent on STAT1 antagonism. Importantly, RABV is neurotropic and during infection of the CNS, astrocytes are the major IFN producers. As astrocytes are abortively infected they are not subject to efficient viral antagonism of IFN induction [28], so the capacity of RABV to invade neurons pre-activated by paracrine signaling is expected to be critical to disease. However, the potential importance of P protein/STAT1 antagonism in the disarming of an established antiviral state, and its relationship with pathogenesis, has not been examined.

Here, we assessed the replication of CE(NiP) and Ni-CE in IFN pre-activated cells, finding that, while replication is initially suppressed, CE(NiP), but not Ni-CE, progressively recovered, indicating roles for P protein in overcoming an established antiviral state. Significant IFN signaling was detectable > 12 h post-IFN treatment, correlating with a persistent, low level of pY-STAT1. The signaling was strongly inhibited by Ni P protein through progressive cytoplasmic accumulation of pY-STAT1, but Ni-CE P protein was defective for this antagonistic function. Thus, it appears that following initial IFN activation, prolonged phosphorylation/dephosphorylation cycling of STAT1 persists to maintain an antiviral state. By recruiting pY-STAT1 into inactive complexes *via* P protein, pathogenic RABV progressively deactivates the antiviral state. In contrast, Ni-CE P protein binds pY-STAT1, but does not effect efficient cytoplasmic retention, resulting in prolonged signaling that correlates with viral attenuation.

## Methods

### Plasmids and cell culture

Constructs to express wild-type and mutated Ni and Ni-CE P proteins and CVS-N protein are described elsewhere [8,16,25,46]. Plasmids used for the luciferase reporter gene assays were pISRE-luc (Stratagene) and pRL-TK (Promega).

Human neuroblastoma SK-N-SH cells (ATCC number HTB-11) and mouse neuroblastoma C1300 (NA) cells [47] were maintained in Eagle’s minimal essential medium supplemented with 10% foetal calf serum. Lipofectamine 2000 (Invitrogen) was used for plasmid DNA transfection into SK-N-SH cells. HEK293T and COS-7 cells were maintained in Dulbecco’s minimal essential media supplemented with 10% foetal calf serum. Transfections were performed using Lipofectamine 3000 (Invitrogen).

### Viruses

RABV strains Ni and Ni-CE used in this study (Fig 1A) were previously recovered from cloned cDNA [44,48]. The chimeric virus CE(NiP), which has the Ni P gene in the Ni-CE genome background (Fig 1A) was generated by a reverse genetics approach [44]. Ni-CE and CE(NiP) strains expressing firefly luciferase (Ni-CE-Luc and CE(NiP)-Luc, respectively) (Fig 1B) were established in our previous study [49]. Stocks of all RABV strains were propagated in NA cells and stored at -80°C until use.

### RABV infection assays

To assess the effect of multiplicity of infection (MOI) on the sensitivity of RABV to IFN, SK-N-SH cells were inoculated with Ni-CE or CE(NiP) at MOI of 0.01 or MOI of 2 and then were cultured in growth medium with or without 500 U/ml of human IFN-α2a (PBL Interferon Source). Supernatant was collected at 3 or 2 days post-inoculation (dpi) for cultures infected at MOI of 0.01 or MOI of 2, respectively, and stored at -80°C. Virus titers were determined as reported previously [25].

**Fig 2:**
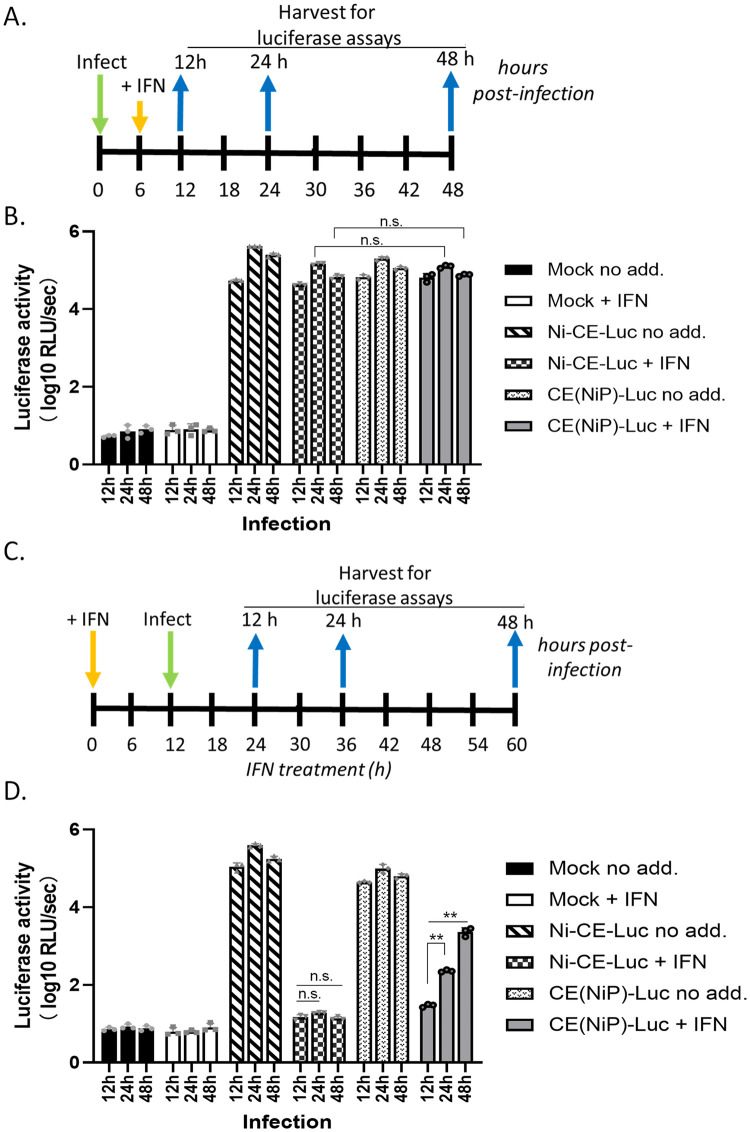
Ni-CE and CE(NiP) viruses are resistant to IFN signaling in infected cells but differ in replication in cells pre-activated by IFN. (A) and (C) show schematics of timelines for experiments shown in (B) and (D), respectively. (A, B) SK-N-SH cells were infected with the indicated virus (MOI of 3), or mock infected, for 6 h before incubation in media without (no add.) or with IFN (+ IFN) for the indicated times post-infection, and harvesting of cells for measurement of luciferase activity. (C, D) SK-N-SH cells were incubated with or without IFN for 12 h before infection with the indicated virus (MOI of 3), or mock infection, for the indicated times, and harvesting for measurement of luciferase activity. Results of luciferase assays (B, D) show mean with SD (n = 3). n.s., p > 0.05; **, p ≤ 0.01.

To examine the effect of IFN treatment post-infection on RABV replication, SK-N-SH cells were inoculated with Ni-CE-Luc or CE(NiP)-Luc at MOI of 3 and then cultured in growth medium for 5 hours. At 6 hours post-inoculation (hpi), the medium was replaced with growth medium with or without 500 U/ml of universal type I IFN (IFNα; PBL Interferon Source). The cells were lysed at 12, 24 and 48 hpi and subjected to the luciferase assay using the Luciferase Assay System (Promega) (see timeline in Fig 2A). To examine the effect of IFN treatment before infection on RABV replication, SK-N-SH cells were incubated with or without 500 U/ml of IFNα for 12 hours before inoculation with Ni-CE-Luc or CE(NiP)-Luc at MOI of 3 for 1 h. After the inoculation, the cells were incubated again in growth medium for 12, 24 and 48 hpi (see timeline in Fig 2C); with the exception of the virus adsorption (for 1 h), the incubation of cell samples in media either with or without 500U IFNα was continuous throughout the experiment (Fig 2C). Following incubation, cells were harvested for analysis by luciferase assay (Fig 2C).

### Luciferase reporter gene assays

**Fig 3:**
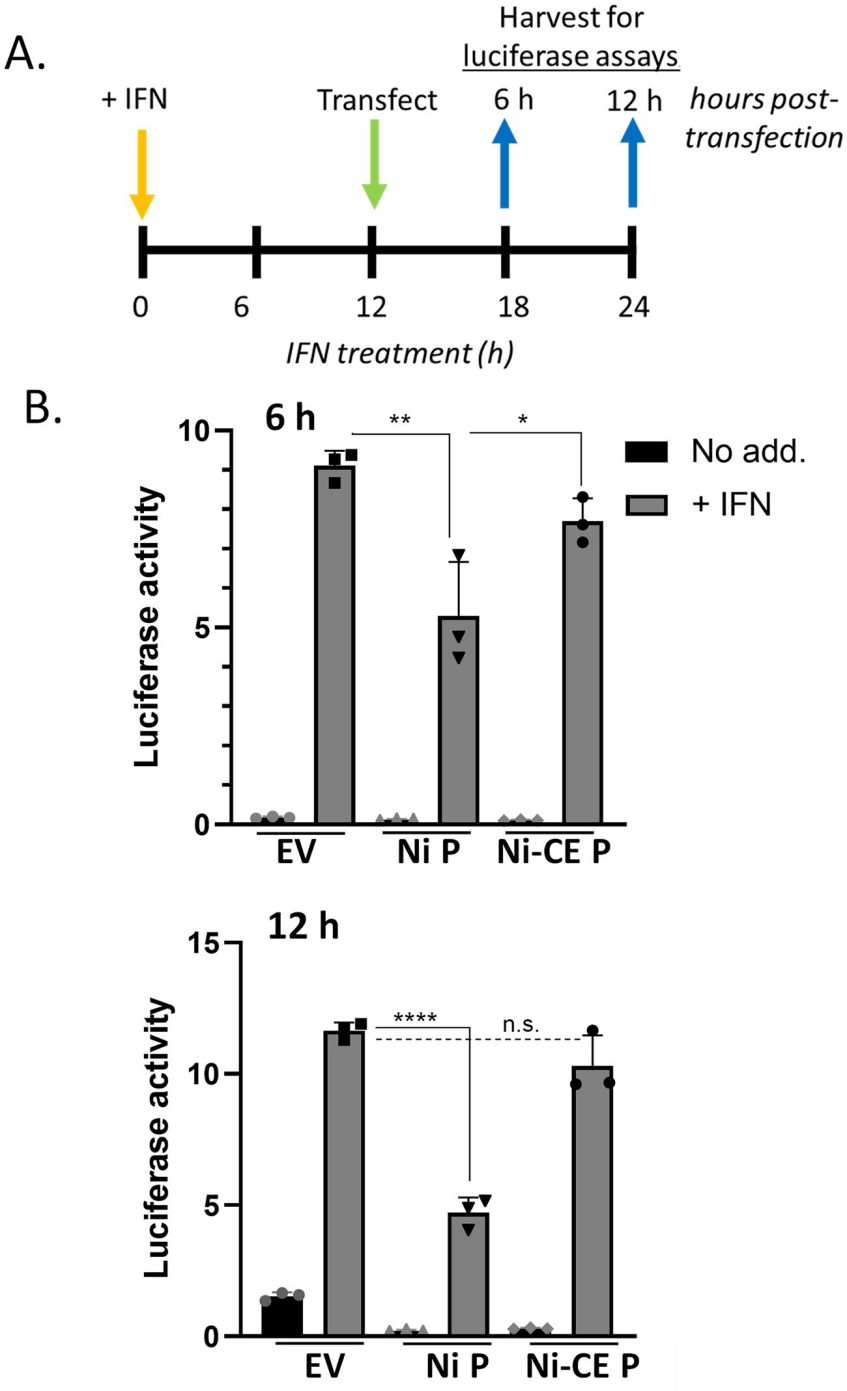
IFN/STAT1 signaling persists for over 12 h and is differentially inhibited by expression of Ni and Ni-CE P proteins. (A) Timeline of experiments performed in (B); SK-N-SH cells were treated with or without IFN for 12 h before transfection with plasmid to express Ni or Ni-CE P protein, or empty vector control (EV), together with plasmids for the IFN/STAT1-dependent luciferase reporter assay; luciferase activity was measured 6 h or 12 h later. (B) Results of luciferase assays show mean with SD (n = 3). n.s., p > 0.05; *, p ≤ 0.05; **, p ≤ 0.01; ****, p ≤ 0.0001.

**Fig 4:**
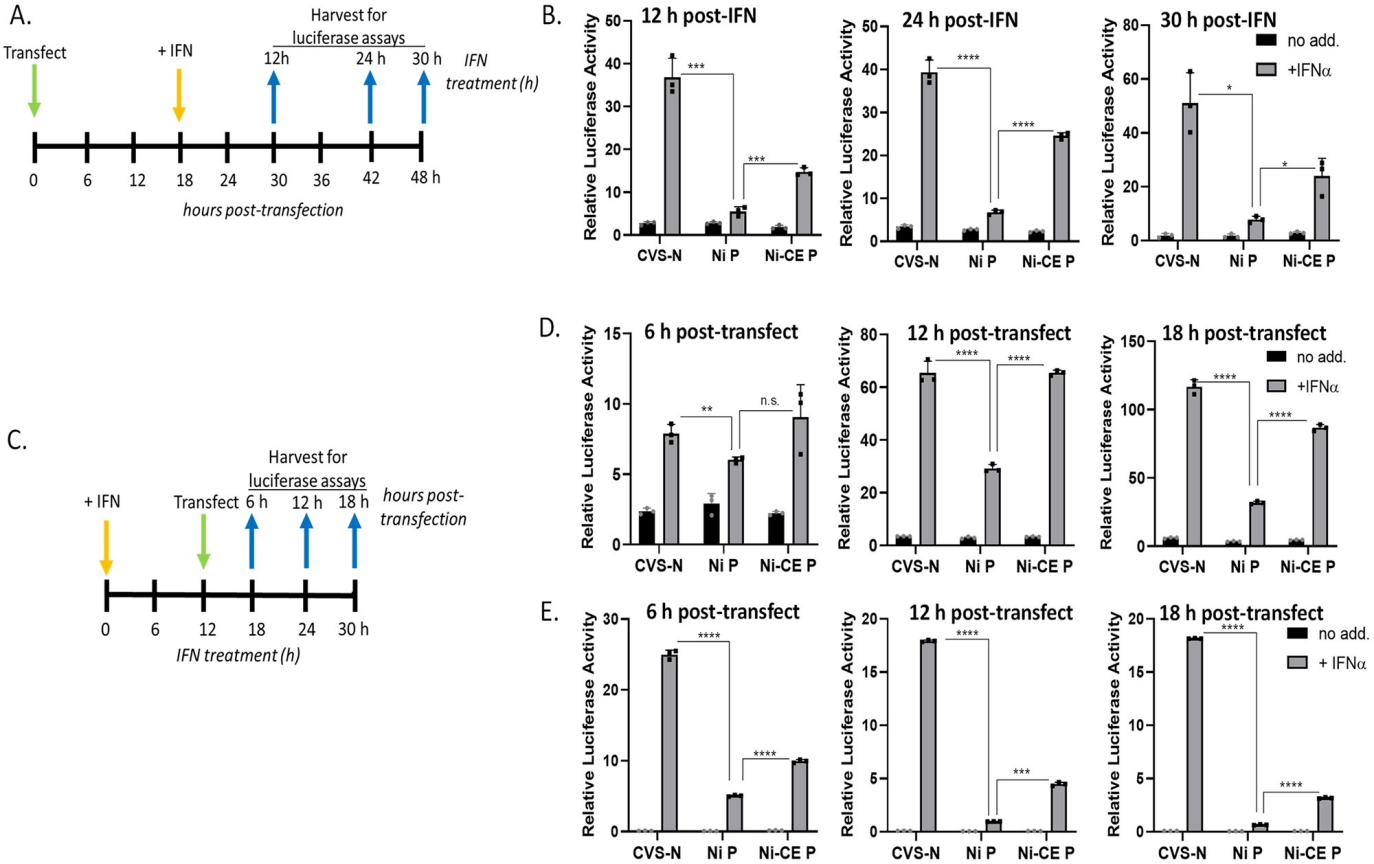
Ni-CE P protein is defective for antagonism of persistent IFN/STAT1 signaling. (A) Timeline of experiments performed in (B); COS-7 cells were transfected with plasmids to express Ni P protein, Ni-CE P protein, or CVS-N protein (control) fused to the C-terminus of GFP, together with plasmids for the IFN/STAT1-dependent luciferase reporter assay, followed by treatment with IFN at 18 h and harvesting of cell samples for luciferase assays at the indicated times. (B) Results of dual luciferase assays, showing mean with SD (n = 3). (C) Timeline of experiments performed in (D) and (E); COS-7 (D) or HEK293T (E) were incubated in media without or with IFN for 12 h before transfection with plasmids to express GFP-fused Ni or Ni-CE P proteins or CVS-N protein, together with plasmids for the luciferase reporter assay; cells were then harvested at the indicated time points. Results of luciferase assays using (D) COS-7 and (E) HEK293T cells showing mean with SD (n = 3). n.s., p > 0.05; *, p ≤ 0.05; **, p ≤ 0.01; ***, p ≤ 0.001; ****, p ≤ 0.0001.

To assess antagonism of IFN signalling induced following establishment of viral protein expression (analogous to autocrine signalling following a viral infection), COS-7 cells were transfected in triplicate with pRL-TK (40 ng), which expresses *Renilla* luciferase constitutively, pISRE-luc (250 ng), which expresses firefly luciferase under the control of an ISRE-containing promoter, and plasmid to express the indicated viral protein or empty vector (EV) (250 ng). 18 h post-transfection, cells were incubated in media with or without 1000 U/ml IFNα (PBL Interferon Source) for 12, 24 or 30 h. To assess IFN signalling activity and antagonism by viral proteins expressed in cells following establishment of an antiviral state (analogous to infection of cells following activation by paracrine signalling), COS-7 or HEK293T cells were treated with 1000 U/ml IFNα for 12 h before transfection as above and incubation for a further for 6, 12 or 18 h. Experiments using SK-N-SH cells to assess IFN signalling and antagonism by viral proteins expressed following establishment of an antiviral state (analogous to paracrine signalling) were performed similarly to those above except that cells were treated with or without 500 U/ml IFNα. Following the indicated incubation periods (see timelines in Figs 3A, 4A and 4C), cells were lysed using passive lysis buffer (Promega) and lysates were subjected to a dual luciferase assay as previously described [16,50]. Values for firefly luciferase activity were normalised to those for *Renilla* luciferase by calculating the ratio of firefly to *Renilla* luminescence.

### Western blot analysis and immunoprecipitation

**Fig 5:**
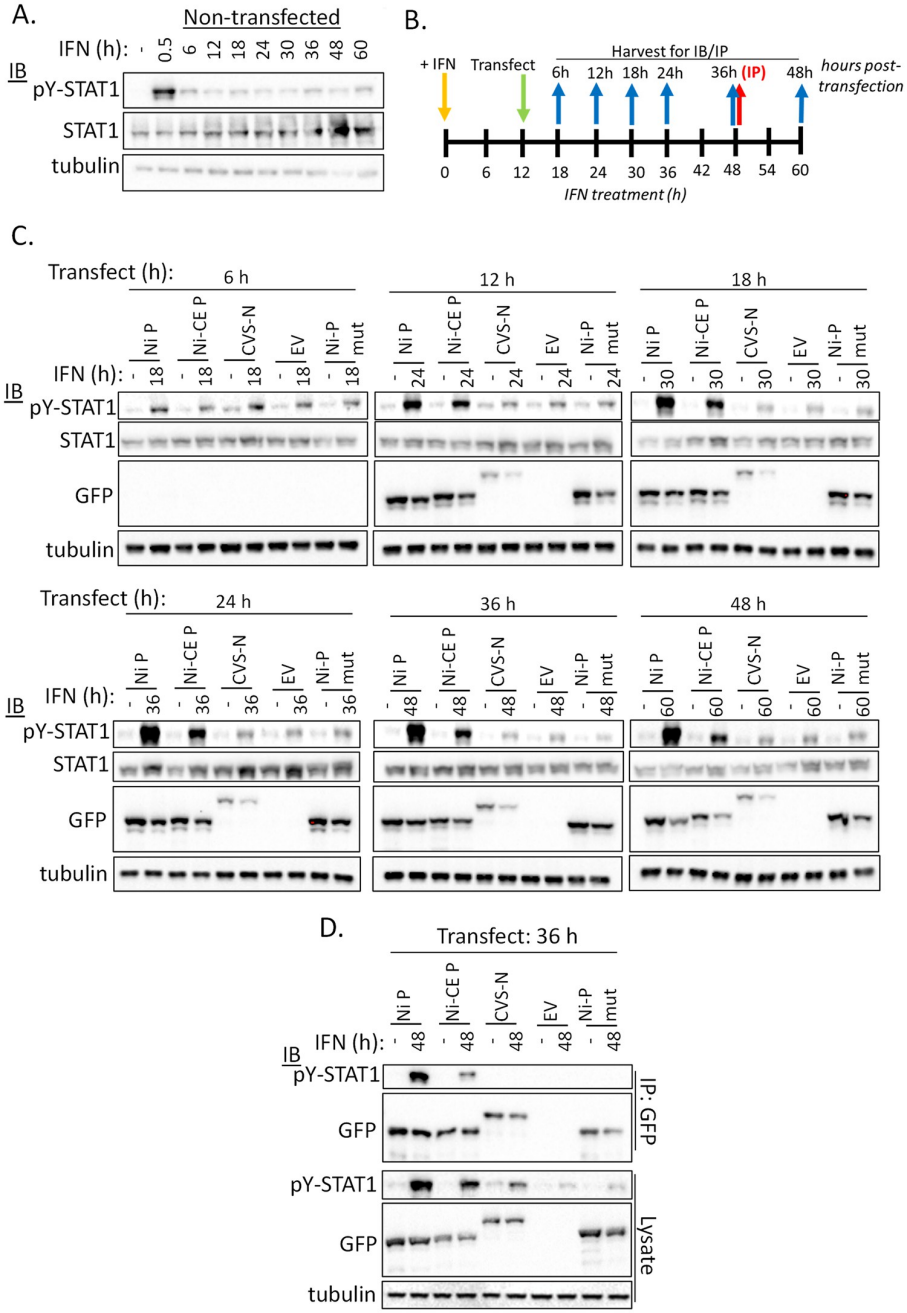
P protein binds and accumulates residual pY-STAT1 in IFN pre-treated cells. (A) Non-transfected COS-7 cells were treated with IFN for the indicated periods before lysis and analysis by immunoblotting (IB) using the indicated antibody. (B) Timeline of experiments shown in (C) and (D); COS-7 cells were incubated in media with or without IFN and transfected 12 h later with empty vector (EV) or plasmids to express the indicated GFP-fused proteins (C, D); cells were harvested at the indicated times for analysis of lysates by IB (blue arrows), or immunoprecipitation for GFP (IP, red arrow). (C, D) IB analysis of cell lysates (C) and IPs (D) using the indicated antibodies.

To assess STAT1 phosphorylation/dephosphorylation following IFN treatment, non-transfected COS-7 cells growing in 12 well plates were treated with 1000U IFNα for periods from 0.5 to 60 h. To examine the effects of P protein expression on IFN-activated STAT1 phosphorylation, COS-7 cells were incubated in media containing 1000U IFNα, or without IFN α, for 12 h before transfection with empty vector (EV), or with plasmid to express Ni or Ni-CE P protein, Ni P protein mutated to substitute F209 and D235 for alanine (Ni-P mut), or CVS N protein (1 μg plasmid per well), before incubation for periods from 6 h– 48h (see schematic timeline in Fig 5B); the incubation in media either with or without IFNα was continued throughout the experiment. Following incubation, cells were washed with PBS and harvested and lysed in lysis buffer (25mM Tris, 150mM NaCl, 0.2 mM EDTA, 0.5% IGEPAL; supplemented with cOmplete protease inhibitor and PhosSTOP). Cleared lysates were separated by SDS-PAGE and transferred to a nitrocellulose membrane for immunoblotting analysis using rabbit anti-pSTAT1 (CST #9167), rabbit anti-STAT1 (CST #14994), mouse anti-GFP (Abcam Ab6556) or mouse anti-tubulin antibody (Sigma T8328), with either goat anti-rabbit HRP-conjugated or goat anti-mouse HRP-conjugated secondary antibodies, before visualisation using ECL Lightening Plus reagent.

To directly assess the interaction of viral proteins with pY-STAT1, COS-7 cells growing in 6 well plates were treated with 1000U IFNα for 12 h before transfection to express the protein of interest (3 μg plasmid per well). Cells were lysed 36 h post-transfection as above (see timeline in Fig 5B). A portion of lysate was retained for analysis by western blotting and the remainder was subjected to immunoprecipitation using GFP-Trap (Chromotek #gmta-20) according to the manufacturer’s instructions. Immunoprecipitate and lysate were analysed by western blotting as above.

### Immunofluorescence staining and confocal laser scanning microscopy (CLSM)

**Fig 6:**
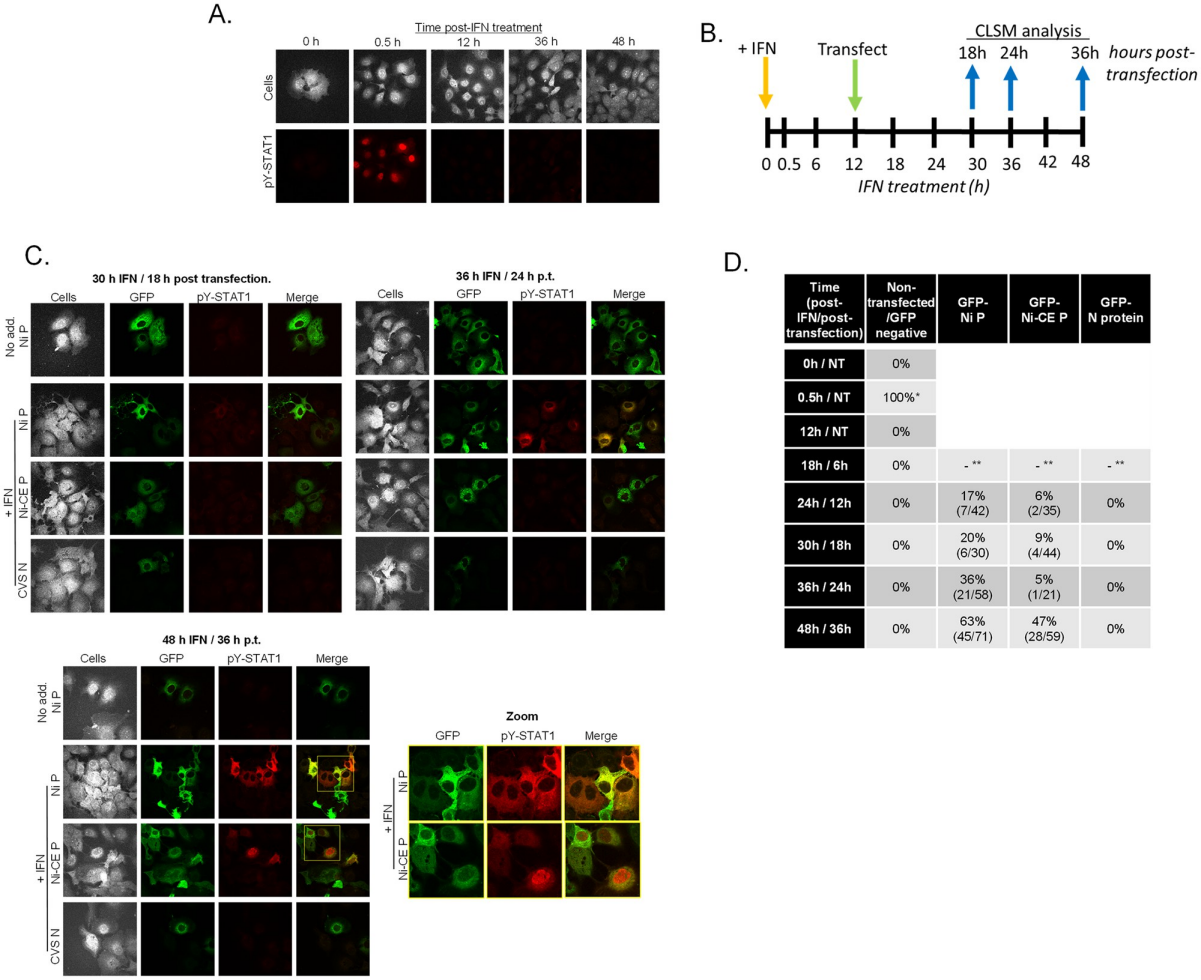
Ni and Ni-CE P proteins cause accumulation of pY-STAT1 in different subcellular sites in IFN pre-treated cells. (A) Non-transfected COS-7 cells were treated with IFN for the indicated periods before fixation and immunostaining for pY-STAT1 and analysis by CLSM. (B) Timeline for experiment shown in (C); COS-7 cells treated with IFN for 12 h were transfected with plasmids to express GFP-fused Ni P protein, Ni-CE P protein or CVS-N protein, before fixation and immunostaining at the indicated times (blue arrows) for CLSM analysis. (C) Representative CLSM images are shown; boxes in yellow are expanded in the zoom panel; identical microscope settings were used for all samples. Non-transfected cells or cells lacking GFP expression (‘Cells’ panels in A, C) were visualized by enhancement of image brightness to detect auto-fluorescence. (D) Summary of results from CLSM analysis showing the percentage of cells with detectable pY-STAT1 labelling; analysis included non-transfected cells (experiment shown in A), and GFP-positive and GFP-negative cells in samples transfected with plasmid to express Ni P, Ni-CE P or N protein (experiment shown in C). * Data are from > 15 fields of view (capturing > 100 cells) for each condition; NT (non-transfected); **, GFP not detectable. For values of 0%, pY-STAT1 was not detected in 15 fields of view (> 100 cells).

To assess pY-STAT1 activation and localization following IFN treatment, COS-7 cells growing in a 12 well plate on coverslips were treated with 1000U IFNα for periods from 0–48 h. To assess effects of viral proteins on pY-STAT1 in cells pre-treated with IFN, cells were treated as above for 12 h before transfection to express proteins of interest, and incubation for a further 12, 18, 24 or 36 h (see timeline in Fig 6B). Following incubations, cells were fixed in 4% formaldehyde (15 min, room temperature) before permeabilization in ice-cold 100% methanol (10 min, -20°C). Cells were then blocked (1 x PBS (pH 7.4), 5% BSA, 0.3% Triton X-100) before incubation with anti-pSTAT1 (CST #9167, 1/800 dilution in blocking buffer, 2 h, room temperature) followed by anti-rabbit Alexa Fluor 568. Cells were mounted in Mowiol mounting medium and imaged by CLSM using a Nikon C1 Inverted confocal microscope with 63x oil objective (Monash Micro Imaging, Monash University). Digitised images were processed using Fiji software (NIH).

### Statistical analysis

Unpaired two-tailed Student’s t-test was performed using Prism software (version 8, GraphPad).

## Results

### CE(NiP) but not Ni-CE virus can replicate in cells pre-activated by IFN

We previously found that infection of cells with Ni, CE(NiP) and Ni-CE virus at MOI of 0.01, and incubation in media containing IFN following virus adsorption results in stronger inhibition of replication of Ni-CE virus than the viruses expressing Ni P protein [25]. Similar experiments using CE(NiP) virus mutated to prevent P protein-STAT1 interaction indicated that infection at MOI of 0.001 followed by IFN treatment at 1 day post-infection (dpi) resulted in substantially decreased titers of the mutant virus [32]. In both cases, the reduced resistance to IFN correlated with attenuation, indicating that pathogenesis depends on IFN/STAT1 antagonism. However, since the MOIs used will result a mixed population of infected and non-infected cells, the differences following IFN treatment could relate to suppression of replication in infected cells (analogous to autocrine signaling) and/or suppression of replication of progeny virus following invasion of pre-activated cells (analogous to paracrine signaling).

To investigate these possibilities, we examined the impact of MOI on replication following IFN treatment by infecting SK-N-SH cells with Ni-CE or CE(NiP) virus at MOI of 0.01 or MOI of 2. After virus adsorption, the cells were incubated with or without IFN (i.e. in media containing recombinant IFN, or with no IFN added) (Fig 1C and 1D). In the MOI of 0.01 condition, only a small proportion of cells will be infected before incubation with IFN, while in the MOI of 2 condition, most or all cells will be infected. Thus, replication in the former condition involves (1) viral antagonism of IFN signaling in infected cells (mimicking autocrine signaling) and (2) subversion of an antiviral state established in non-infected target cells before invasion by progeny virus (mimicking paracrine signaling). In contrast, in the latter condition, replication is primarily dependent on viral antagonism of IFN signaling in infected cells (autocrine signaling). By using viruses expressing P proteins with differing STAT1 antagonist function and pathogenicity (Fig 1A), this experiment also assessed the role of P protein in replication under the different conditions, and its potential relationship to disease. Titers were measured after 3 dpi (for MOI of 0.01) or 2 dpi (for MOI of 2); the measurement at 2 dpi for the higher MOI is necessitated due to strong cytopathic effects of the viruses by 3 dpi.

In the MOI of 0.01 experiment, production for both viruses was inhibited by IFN, but Ni-CE virus was substantially more sensitive to IFN than CE(NiP), indicated by a decrease in titer of Ni-CE virus by 5.2 log following addition of IFN, whereas the titer of CE(NiP) was reduced by only 3.0 log (Fig 1C). In contrast, in the MOI of 2 experiment, IFN had minimal effects on the replication of either virus, with little difference observed between the different viruses (titers reduced by only 1.1 and 0.7 log for Ni-CE and CE(NiP), respectively, following IFN treatment) (Fig 1D). These data are consistent with RABV being relatively resistant to the production of IFN-dependent antiviral mechanisms in cells where an active infection is established, but susceptible to a pre-established antiviral state, such that spread into pre-activated cells following low MOI infection is inhibited. Importantly, the greater sensitivity to IFN of Ni-CE virus compared with CE(NiP) virus at low MOI (Fig 1C) suggested a major difference in the capacity to surmount an established antiviral state, due to differing P protein function. However, the less potent STAT1-antagonist function of Ni-CE P protein compared with Ni P protein [25] appears sufficient to effectively suppress the establishment of an antiviral state by IFN signaling in infected cells (Fig 1D).

To directly assess viral replication in cells treated with IFN pre- or post-infection, we used CE(NiP) and Ni-CE virus engineered to express luciferase through insertion of a luciferase gene in the G-L intergenic region [49] (Fig 1B), which enables measurement of primary and secondary transcription. SK-N-SH cells were infected at high MOI (MOI of 3) for 6 h before treatment with or without IFN by incubation in media containing 500 U/ml recombinant IFN or media with no IFN added (Fig 2A and 2B); alternatively, cells were infected at MOI of 3 following a 12 h incubation in media with or without IFN (Fig 2C and 2D). The incubation of cells either with or without IFN was continued for the remainder of the experiment. The first condition, in which cells are infected before incubation with IFN, assesses effects of IFN on an established infection (similar to autocrine signaling), and the second condition, in which cells are incubated with IFN before infection, assesses effects of an established antiviral state on subsequent infection (similar to infection of cells previously activated by paracrine signaling).

Consistent with the experiments using different MOIs (Fig 1C and 1D), treatment following establishment of infection did not substantially affect transcription/replication and there was no significant difference between the luciferase activity in Ni-CE and CE(NiP)-infected cells at 24 or 48 h post-infection (Fig 2B). In IFN pre-treated cells, there was a strong antiviral effect at 12 h post-infection, indicated by clearly reduced luciferase activity of both viruses compared to that in non-treated cells (Fig 2D). However, luciferase activity increased in CE(NiP)-infected cells at 24 h and 48 h. In contrast, there was no significant increase in luciferase activity in Ni-CE-infected cells (Fig 2D). This indicated that IFN treatment induces a persistent antiviral state that prevents replication by the attenuated Ni-CE virus for up to 60 h post-treatment (infection for longer periods was not possible due to cytopathic effects of infection). While initially impeded by the antiviral state, CE(NiP) virus can progressively reverse this state, enabling transcription/replication to recover (Fig 2D).

Taken together, these data indicate that the residual IFN antagonist activity of Ni-CE P protein is sufficient to suppress IFN signaling and the emergence of an antiviral state in pre-infected cells, but not to reverse an established antiviral state in IFN-pretreated cells. Thus, differences in P protein, which correlate with pathogenesis, relate to viral ‘disarming’ of the antiviral state.

### IFN/STAT1 signaling persists over extended periods and is differentially antagonized by Ni and Ni-CE P protein

The differing replication of CE(NiP) and Ni-CE in IFN pre-activated cells indicated that the Ni and Ni-CE P proteins differ in a mechanism to inactivate the antiviral state. The capacity of virus to replicate in IFN-activated cells is likely to depend at least in part on subversion of antiviral functions of certain IRGs [22]. However, while P protein interacts with PML and can affect PML nuclear body structures [39], specific functions of P protein in antagonizing anti-RABV effector functions of PML or other IRGs have not been reported, to our knowledge. Since CE(NiP) and Ni-CE viruses, and P proteins thereof, differ in antagonism of IFN/pY-STAT1 signaling in pre-infected cells or cells pre-transfected to express P protein [16,25], we considered that this might be an important mechanism in disarming of a pre-established antiviral state by RABV. Such a mechanism appears contrary to conventional understanding that pY-STAT1 is dephosphorylated and inactivated within hours of IFN treatment, but extended STAT1-dependent IRG transcription has been reported [9,10,20]. Thus, we hypothesized that residual pY-STAT1 might support persistent signaling to maintain the antiviral state, so that antagonism of pY-STAT1 would progressively disable the response, with the activity of Ni P protein superior to that of Ni-CE P protein.

We examined IFN-I/STAT1/2 signaling using an ISRE-dependent luciferase reporter gene assay as previously described [8,16,25,30,32]. We previously used ‘conventional’ assays where cells are treated with IFN following transfection with luciferase plasmids and either co-transfection with plasmids to express viral proteins, or infection by virus; this approach showed that Ni-CE P protein is less able to inhibit IFN/STAT1 signaling than Ni-P protein, in transfection and infection systems [25]. Importantly, for the current study we included a different approach where cells were treated with IFN for 12 h before transfection of luciferase constructs and viral protein expression constructs, and subsequent measurement of luciferase activity (Figs 3A and 4C). As the luciferase plasmids only report transcription after transfection, these assays will only measure signaling persisting after 12 h IFN treatment. Because Ni-CE virus transcription/replication (and, hence, P protein expression) is prevented by pre-treatment with IFN (Fig 2D), we used transfection rather than infection to compare effects of Ni-CE and Ni P protein on persistent IFN/STAT signaling.

In SK-N-SH cells treated with IFN for 12 h before transfection for 6 h or 12 h, substantial luciferase activity was detected compared with untreated cells, indicative of persistent signaling (Fig 3B). At 6 h post-transfection, Ni P protein moderately suppressed IFN signaling, with much greater inhibition apparent at 12 h post-transfection (c. 60% reduction), while Ni-CE P protein had little to no effect (Fig 3B). As P protein selectively targets pY-STAT1, these data suggest that the persistent signaling is due, at least in part, to pY-STAT1, which is strongly antagonized by Ni P protein, with Ni-CE P protein partially defective [25]. Thus, the extended antiviral state appears to involve prolonged pY-STAT1 signaling, with the differing recovery of replication by CE(NiP) and Ni-CE viruses in IFN pre-activated cells (Fig 2D) relating to a differing capacity to antagonize this signaling.

To confirm these results and enable further mechanistic analysis, we repeated the analysis using COS-7 cells transfected to express GFP-fused P proteins or GFP-fused to the nucleoprotein of challenge virus standard strain RABV (CVS N protein, which does not bind or antagonize to STAT1 and represents a standard control), as previously used for analysis of RABV/STAT1 interactions [8,16,25,32,37]. Assays of cells transfected prior to IFN treatment (Fig 4A) confirmed a substantial inhibition of IFN signaling by Ni P protein, with the antagonistic function of Ni-CE P protein clearly reduced (Fig 4 B).

In cells pretreated with IFN for 12 h before transfection (Fig 4C and 4D), persistent IFN/STAT signaling was evident, and this was inhibited by Ni P protein at all times post-transfection, while antagonism by Ni-CE P protein was reduced (Fig 4D). Similar analysis of P proteins in HEK293T cells also indicated persistent signaling, which was inhibited by Ni P protein, with a significant impairment in antagonistic function of Ni-CE P protein (Fig 4C and 4E). Thus, IFN/STAT1 signaling persists beyond the expected time for pY-STAT1 dephosphorylation/inactivation [3,5,19], and differential antagonism of this signaling by Ni and Ni-CE P proteins correlates with the capacity of virus expressing these proteins to replicate in cells pre-activated by IFN (Fig 2C and 2D).

### Residual levels of pY-STAT1 persist over extended periods in IFN-activated cells

The above data indicated that, following initial activation and dephosphorylation of STAT1, a level of pY-STAT1-dependent signaling persists and is required to maintain an antiviral state. Although previous studies indicate that pY-STAT1 is rapidly depleted following the activation peak, a number of reports indicate persistence of low levels of pY-STAT1 exceeding those in non-treated cells, after extended IFN treatment (> 48 h; e.g. [4,9,11,14,51,52]). We thus hypothesized that some pY-STAT1 is retained following depletion, providing signaling sufficient to support ongoing antiviral functions. Western blot analysis of pY-STAT1 in lysates of COS-7 cells indicated that, as expected, pY-STAT1 was at low or undetectable levels in untreated cells, but was strongly induced after 0.5 h IFN treatment (Fig 5A) [4,7–9,11,15,16]. Consistent with reports that pY-STAT1 is dephosphorylated from c. 1–3 h post-IFN treatment [4,7,9,11,15], pY-STAT1 was clearly reduced at 6 h post-IFN treatment and subsequent time points (Fig 5A). Nevertheless, residual pY-STAT1 was detectable and persisted for at least 60 h post-treatment (Fig 5A); the signal for STAT1 also appeared to increase at later time points, possibly relating to the fact that STAT1 is an IRG and so inducible by IFN [10]. These data indicate that the persistent signaling in reporter assays likely involves residual pY-STAT1, consistent with the observed inhibitory effect of P protein, a selective pY-STAT1 antagonist.

### Expression of P protein in cells pre-activated by IFN causes accumulation of pY-STAT1

The above data are consistent with persistent IFN signaling in activated cells occurring through dynamic phosphorylation/dephosphorylation cycling of a fraction of cellular STAT1 to maintain an extended antiviral state. The alternative possibility is that a small population of pY-STAT1 is retained, but does not cycle dynamically and somehow maintains ongoing transactivation function. Because P protein inhibits STAT1 dephosphorylation [8,16,17], it provides a tool to examine these possibilities and so elucidate mechanisms of the innate immune response: specifically, if STAT1 is undergoing phosphorylation/dephosphorylation cycles, inhibition of the latter by expression of P protein should result in accumulation of pY-STAT1.

To confirm that P protein interacts with the residual pY-STAT1 in cells following prolonged IFN treatment, we analyzed pY-STAT1 in cells treated with IFN for 12 h before transfection to express P proteins and controls (Fig 5B); the controls included transfection with empty vector (EV) or with plasmids to express CVS-N protein, or Ni-P protein mutated to disable the STAT1-binding site (F209A/D235A, Ni-P mut [8]). Western blot analysis of cell lysates indicated that at 6 h post-transfection, little P protein expression was evident and levels of pY-STAT1 were similar in all IFN-treated samples (Fig 5C). However, at 12 h post-transfection and subsequent time points, P protein expression was detected, and correlated with accumulation of pY-STAT1 in cells expressing Ni and Ni-CE P proteins (Fig 5C). These data are consistent with the finding that both proteins bind to STAT1 [25], and we confirmed an interaction with pY-STAT1 at 48 h post-IFN treatment (36 h post-transfection) by immunoprecipitation of P protein (Fig 5B and 5D). pY-STAT1 levels in cells expressing controls were clearly reduced compared with those in cells expressing Ni or Ni-CE P proteins, and no interaction with pY-STAT1 was evident for any of the controls.

These findings indicate that phosphorylation/dephosphorylation of STAT1 is ongoing at steady state to maintain a low level of pY-STAT1. Binding by P protein recruits pY-STAT1 into inhibitory complexes, which are not efficiently dephosphorylated, while ongoing phosphorylation generates more pY-STATs that are subsequently recruited. Of note, although Ni-CE P protein caused accumulation of pY-STAT1, the level of pY-STAT1 in lysates and amount of pY-STAT1 co-precipitated by Ni-CE P protein was reduced compared with that observed for Ni P protein. Thus, Ni-CE P protein is less able to retain pY-STAT1 in inactive complexes, correlating with defective antagonism.

### Ni and Ni-CE P protein accumulate pY-STAT1 in different subcellular locations

To assess accumulation of pY-STAT1 in the cellular context, we treated COS-7 cells with or without IFN for various time periods before fixation and immunostaining for pY-STAT1, and analysis by CLSM (Fig 6). As expected, in non-transfected cells, pY-STAT1 was not detected in untreated cells but became apparent in nuclei of c. 100% cells after 0.5 h IFN treatment; following 12–48 h treatment, pY-STAT1 was depleted to below detectable levels (Fig 6A and 6D); this is consistent with the induction and depletion of pY-STAT1 observed in cell lysates (Fig 5A). We next examined the accumulation of pY-STAT1 in cells treated for 12 h before transfection to express Ni- or Ni-CE P proteins or CVS-N protein for different time periods (Fig 6B). In cells transfected to express Ni P protein, pY-STAT1 became detectable in c. 17% of GFP-positive cells at 12 h post-transfection, and increased over subsequent time points to be detectable in 63% of cells at 36 h post-transfection (Fig 6C and 6D). pY-STAT1 was only detected in samples treated with IFN, and in these samples was observed only in cells expressing GFP-Ni-P protein and not in non-expressing cells in the same field of view (Fig 6C). pY-STAT1 also accumulated in cells expressing Ni-CE P protein, but was less readily detected (c. 6% and 47% of cells at 12 h and 36 h post-transfection, respectively) (Fig 6C and 6D). Importantly, in Ni P protein-expressing cells with detectable pY-STAT1, the proteins colocalized almost exclusively in the cytoplasm, whereas in Ni-CE P protein-expressing cells, significant nuclear localization of pY-STAT1 was observed (Fig 6C, zoom panel). Thus, it appears that both proteins interact with pY-STAT1 in IFN pre-activated cells and inhibit dephosphorylation, but Ni-CE P protein cannot prevent nuclear localization. This is consistent with results from cells transfected with P protein or infected before IFN treatment [25], which indicated that the differing antagonistic function of Ni and Ni-CE P proteins relates to differing subcellular trafficking of P proteins and, hence, P protein-STAT1 complexes. The defect of Ni-CE P protein in inhibiting STAT1 nuclear accumulation and, consequently, transactivation function would be expected to increase dephosphorylation and recycling of STAT1, which is likely to account for the apparent reduction in pY-STAT1 in cells expressing Ni-CE P protein compared with Ni P protein (Fig 5C). Together, these defects in Ni-CE P protein result in impaired inhibition of persistent IFN signaling, correlating with viral attenuation.

## Discussion

In this study, we have characterized a mechanism by which RABV can invade and replicate within cells that are in a pre-established antiviral state, finding that targeting of pY-STAT1 is a critical element in inactivation of the antiviral condition. Central to this is the observation that maintenance of the extended antiviral state following IFN activation is dependent on persistent residual signaling by STAT1. This includes a significant contribution by a fraction of STAT1 that appears to be undergoing dynamic phosphorylation/dephosphorylation cycles, consistent with ongoing active signaling, which results in a low steady-state level of pY-STAT1. The data indicate that tyrosine phosphorylation of only a small proportion of STAT1 is required to support IRG transcription, based on the substantial effect of the pY-STAT1-antagonist P protein on signaling in cells pre-activated by IFN (Fig 4D and 4E). Persistent pY-STAT1 stimulation is possibly required to counteract cellular negative regulatory mechanisms [1,3], such that viral inhibition of pY-STAT1 signaling results in a dominance of cellular countermeasures, ultimately deactivating the antiviral state.

**Fig 7:**
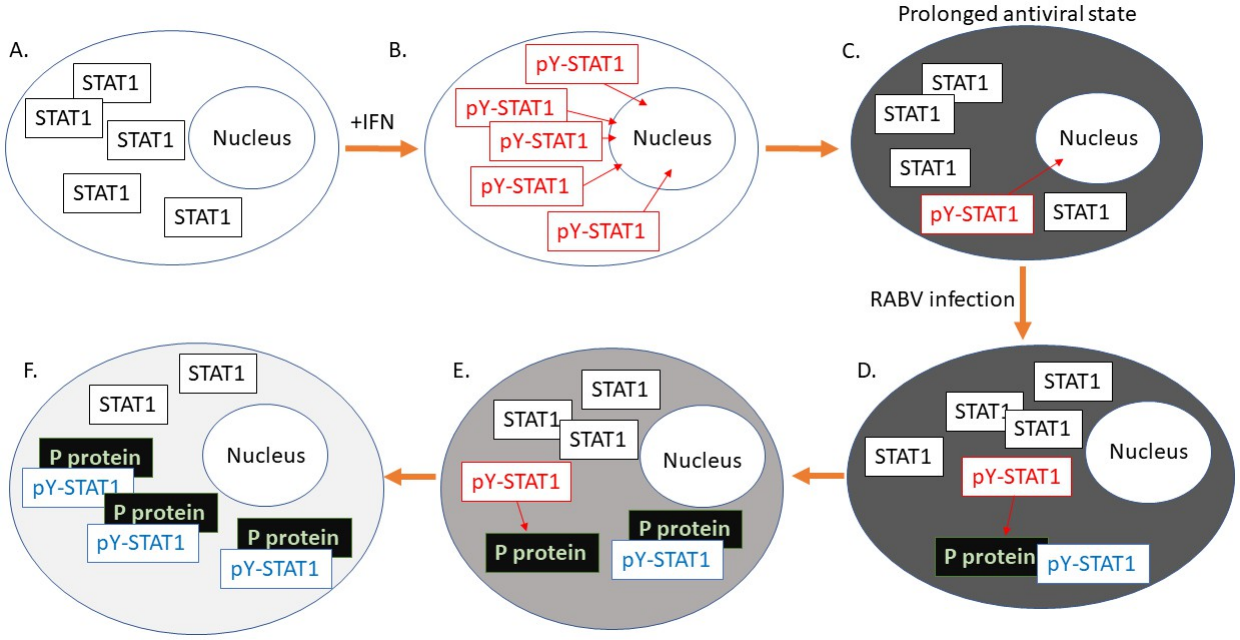
Proposed model for deactivation of the antiviral state by RABV P protein. In non-activated cells (A), STAT1 is largely unphosphorylated (black boxes); following IFN activation, high levels of pY-STAT1 (red boxes) are induced rapidly (B) and regulate the expression of IRGs, resulting in the establishment of an antiviral state (dark grey cell, C). pY-STAT1 is dephosphorylated, but a low residual level of phosphorylation/dephosphorylation cycling and signaling by pY-STAT1 persists, contributing to the maintenance of a prolonged antiviral state (C). Following infection by RABV, P protein targets pY-STAT1 recruiting it to inactive cytoplasmic complexes (blue box, D); low levels of STAT1 activation continue, resulting in progressive accumulation (E) of pY-STAT1 by P protein, inhibiting persistent signaling to ultimately inactivate the antiviral state (F).

At the molecular level, it appears that, following infection of pre-activated cells, P protein of pathogenic RABV specifically targets the small population of pY-STAT1 and inhibits signaling and dephosphorylation by retaining pY-STAT1 in inhibitory cytoplasmic complexes. The remaining non-phosphorylated STAT1 continues to be activated, and is then recruited by P protein. This progressive accumulation and inhibition of STATs appears to tip the balance between antiviral mechanisms and viral transcription, ultimately enabling the virus to overwhelm the antiviral state (summarized in model shown in Fig 7). P protein of the attenuated Ni-CE strain retains a capacity to bind and accumulate pY-STAT1, but not to effect strong cytoplasmic arrest so that the antagonistic function is impaired. While the residual function of Ni-CE P protein appears sufficient to prevent the activation of strong antiviral effects in cells where infection is established prior to IFN stimulation (Fig 2B), it cannot efficiently surmount a pre-established antiviral state (Fig 2D). The former condition is analogous to initial/early infection of the host and autocrine signaling, where significant expression of Ni-CE P protein, and possibly other viral proteins that contribute to immune subversion [46,53], may be established before substantial activation of STAT1. The latter condition is analogous to a more advanced infection, where paracrine signaling (including from non-infected immune cells), has activated target cells prior to infection, with the weaker antagonistic function and limited expression of Ni-CE P protein by invading virus unable to reverse the antiviral state. In contrast, Ni P protein, that is expressed by pathogenic CE(NiP) virus, is effective in both ‘autocrine’ and ‘paracrine’ conditions due to its greater antagonistic function. The capacity of RABV to overcome an established antiviral state is likely to be particularly important to spread in the central nervous system (CNS), consistent with the attenuation of Ni-CE virus.

Although we detected pY-STAT1 at late time points of IFN treatment, substantial dephosphorylation occurred following the initial pY-STAT1 peak (Fig 5A). The low expression of pY-STAT1 after extended IFN treatment perhaps accounts for the lack of detection of this ‘late’ pY-STAT1 in some studies. IRG expression is also reported to be reduced following an early wave of induction by IFN [5], so it has been assumed that, following initial activation, pY-STAT1 signaling is lost. However, the antiviral response is clearly extended, and this has been attributed to IFN-induced U-STATs [9,10,20]. Our new data support key roles for persistent pY-STAT1 in maintaining the antiviral state, consistent with observations elsewhere of prolonged pY-STAT1 expression [4,9,11,14,51,52]. By showing that a specific inhibitor of pY-STAT1 (P protein) inhibits persistent signaling > 12 h post-IFN treatment, and that this correlates with a reversal of the antiviral state, our data support a substantial role of pY-STAT1, distinct from U-STAT1.

This model, however, does not rule out effects of P protein on U-STAT1. Although the P protein CTD binds to U-STAT1 *in vitro* [8,30], the interaction is of low affinity [8] and significant co-precipitation of P protein and STAT1 from mammalian cells is dependent on IFN, indicating minimal U-STAT1 binding [8,16,17]. Nevertheless, the progressive recruitment of pY-STAT1 into inhibitory complexes will ultimately reduce the pool of ‘by-stander’ U-STAT1. Assuming that inhibition of U-STAT1 is important, this raises the question of why RABV P protein selectively targets pY-STAT1 rather than ‘total’ STAT1 (a mechanism used by a number of other viruses [22]). This may relate to the life cycle of RABV, where incubation can extend to months before retrograde invasion of the CNS and, ultimately, anti-retrograde spread for transmission to new hosts [54,55]. Damage to infected cells would limit retrograde and anti-retrograde spread as well as potentially impairing further transmission by disabling rabid animals; indeed, RABV infection is typically associated with limited sequelae [24]. Specific targeting of pY-STAT1 presumably ensures that STATs are inhibited only as required (during or following IFN activation), minimizing any impact on potential homeostatic functions of U-STATs. pY-STAT1 targeting also ensures efficient deployment of P protein, which has multiple roles in infection [1,24]. Furthermore, while U-STATs activate certain IRG subsets, pY-STATs are expected to have more potent function, and so may present more critical targets. Following infection of an IFN-activated cell, P protein concentration will initially be limiting, so high-affinity binding to a minor but critical pY-STAT1 subpopulation of cellular STAT1 may ‘gain a foothold’ in immune subversion until increased P protein expression permits more broad antagonism. Such selective mechanisms may be particularly important for P proteins and other IFN antagonists that inhibit STATs *via* direct binding/sequestration, and so have a defined stoichiometric interaction, in contrast to viral proteins that use enzymatic mechanisms [22]. Finally, based on our observation that pY-STAT1 can interact with complexes containing RABV P protein and N protein [56], and reports that STATs can accumulate within viral replication bodies [1,57–59], it is possible that binding/accumulation of pY-STATs not only contributes to immune evasion, but also has distinct roles in replication.

## Supporting information

S1 Data Excel spreadsheet containing, in separate sheets, the underlying numerical data for Fig panels 1C, 1D, 2B, 2D, 3B, 4B, 4D, and 4E. (XLSX)
